# Systemic Glycemic Variation Predicts Mortality of Acute Ischemic Stroke After Mechanical Thrombectomy: A Prospective Study Using Continuous Glucose Monitoring

**DOI:** 10.3389/fneur.2022.817033

**Published:** 2022-03-18

**Authors:** Jiangshan Deng, Ling Li, Fengya Cao, Feng Wang, Hongmei Wang, Hong Shi, Li Shen, Fei Zhao, Yuwu Zhao

**Affiliations:** ^1^Department of Neurology, Shanghai Jiao Tong University Affiliated Sixth People's Hospital, Shanghai, China; ^2^Department of Rehabilitation Medicine, Shanghai Eighth People's Hospital, Shanghai, China; ^3^Clinical Research Center, Shanghai Jiao Tong University Affiliated Sixth People's Hospital, Shanghai, China

**Keywords:** glycemic variation, ischemic stroke, mechanical thrombectomy, mortality, continuous glucose monitoring (CGM)

## Abstract

**Objective:**

We investigated the association of glycemic variation with the clinical outcomes of large vessel occlusion (LVO) induced acute ischemic stroke (AIS) after mechanical thrombectomy (MT).

**Methods:**

We recruited consecutive ischemic patients with stroke. Glucose levels were assessed through continuous glucose monitoring in 70 patients with AIS who had undergone MT. Metrics including percentages of time of glucose levels above the range, the hypoglycemic range, and the time within the range, coefficient of variation, standard deviation (SD), mean of daily differences, mean amplitude of glycemic excursion, largest amplitude of glycemic excursion, high blood glucose index, and low blood glucose index. The outcomes of this observational study were in-hospital mortality, neurological improvement during hospitalization, functional independence, and mortality at follow-up (3 months). The associations of the blood glucose metrics with outcomes were analyzed.

**Results:**

The average period of glucose monitoring was 3.5 days, and serum glucose was recorded 728 times after MT for each person. The glycemic variation expressed in SDs was independently associated with in-hospital mortality [odds ratio (OR): 2.8, 95% confidence interval (CI): 1.276–6.145, *p* = 0.01] and the 3-month mortality (OR: 2.107, 95% CI: 1.013–4.382, *p* = 0.046) after adjusting for potential confounders. There was no association of glycemic variation with the 3-month clinical functional independence.

**Conclusions:**

Increased systemic glycemic variation was associated with higher odds of mortality of LVO-AIS after MT.

**Clinical Trial Registration:**

http://www.chictr.org.cn/showproj.aspx?proj=21016, identifier: ChiCTR-OOC-17012378.

## Introduction

Stroke is a leading cause of death and disability worldwide (1). Early reperfusion is an effective therapy for acute ischemic stroke (AIS) (2). For patients with AIS with proximal large vessel occlusion (LVO) of the anterior circulation, mechanical thrombectomy (MT) reduces disability in patients if it is performed within 6 h of the time when the patients were last known to be healthy, and up to 24 h after stroke onset in patients selected using brain perfusion imaging (3, 4). Although the recanalization rate of MT could reach nearly 80%, only around 50% of the patients achieve functional independence (5). Moreover, the mortality rates of patients with AIS, even after MT, remains high in real-world clinical settings (6). There is a need to identify risk factors for adverse outcomes of AIS after MT and to optimize critical care and management strategies for patients with AIS after reperfusion therapy.

Hyperglycemia occurs in 30–40% of patients with AIS (7). It is commonly accepted that hyperglycemia is associated with poor outcomes after stroke (8). However, intensive glucose control during the acute stroke stage failed to improve the outcomes of patients with AIS (9). In diabetes, blood glycemic variation plays a role, in addition to hyperglycemia and hypoglycemia. Glycemic variation considers fluctuations in blood glucose levels and can potentially provide a more comprehensive assessment of glycemic control after stroke (10). Retrospective studies that did not use continuous blood glucose monitoring showed that glycemic variation may have a negative effect on the outcome of AIS after MT (11, 12).

We hypothesized that increased glycemic variation was associated with poor outcomes of LVO–AIS after MT. In this prospective cohort study, a continuous glucose monitoring system (CGMS) was used to record the blood glucose profile in patients with AIS post-MT. The association of blood glycemic variation with outcomes during hospitalization and at the 3-months follow-up was determined in patients with AIS post-MT.

## Methods

### Patients

This was a single-center, prospective follow-up study. This study protocol was carried out according to the recommendations of the Ethics Committee of Shanghai Jiao Tong University Affiliated Sixth People's Hospital and was registered in the Chinese Clinical Trial Registry (accession number: ChiCTR-OOC-17012378). All the subjects gave written informed consent according to the Declaration of Helsinki.

Patients with AIS involving the anterior circulation who were admitted to the Neurology Department of Shanghai Jiao Tong University Affiliated Sixth People's Hospital and who underwent MT were consecutively recruited to reduce bias. We included patients with confirmed AIS involving anterior circulation LVO and who underwent MT according to the guidelines (13). Briefly, for patients with anterior LVO within 4.5 h of stroke onset, intravenous tissue plasminogen activator (0.9 mg/kg weight, Boehringer Ingelheim, Ingelheim am Rhein, Germany) was administered and was bridged with MT therapy. For patients with stroke onset between 4.5 and 6 h, direct MT was performed after performing non-contrast computed tomography (CT) and CT angiography. For patients with stroke onset between 6 and 24 h, MT was performed after multimodel CT evaluation, according to the guidelines (2). We excluded patients with posterior circulation LVO and those who died within 48 h after the operation.

A Solitaire stent (ev3 Neurovascular, Irvine, CA, USA) with an intracranial support catheter for MT was preferred and was used as the first-line technique in our center. The retrieval attempt was repeated up to three times per target artery. If large artery atherosclerosis causing stenosis was confirmed during thrombectomy and reocclusion occurred, a salvage balloon dilation or stent implantation was performed. All patients were prescribed atorvastatin, antiplatelet, or anticoagulation drugs as needed after the procedure.

Data including age; sex; admission dates; weight; height; smoking status; comorbidities; the National Institutes of Health Stroke Scale (NIHSS) score on admission, 24 h, and 7 days after reperfusion; time from onset to recanalization; and modified thrombolysis in cerebral infarction (mTICI) evaluation of the recanalization state of vessels were recorded.

### Definitions

Blood reperfusion was determined using the mTICI grading system. Grades 2b and 3 were defined as good reperfusion states. Intracranial hemorrhage (ICH) transformation was defined as hyperintensity on the CT scan at 24-h post-MT excluded residual contrast medium. Symptomatic ICH (sICH) was defined as its radiologic appearance plus an increase in the NIHSS score of ≥4 points according to the European Cooperative Acute Stroke Study criteria grading (14). Neurological improvement was defined as an NIHSS score of 0 at 7 days or a decrease in the NIHSS score at 7 days of ≥4 points as compared to the NIHSS score on admission. According to the modified Rankin scale (mRS) at 3 months, excellent functional outcomes were defined as an mRS score of 0 or 1, whereas functional independence was defined as an mRS score of 0–2. Diabetes mellitus was diagnosed based on known preexisting diabetes or with an HbA1c value ≥6.5% (48 mmol/mol) tested after admission in the absence of a known diabetes diagnosis (15).

### Continuous Glucose Monitoring System

All recruited patients were equipped with a CGMS (IPro2, Medtronic MiniMed; Medtronic, Dublin, Ireland) and were monitored for 2–4 consecutive days after MT (16). Briefly, the CGMS device was inserted subcutaneously into the lower abdomen of patients within 6 h after MT. Interstitial fluid glucose concentrations were recorded every 5 min throughout the day and were retrospectively calibrated with fingertip blood glucose measurement. The average time from onset to CGMS device implantation was 9.0 ± 6.4 h, and the mean period of glucose monitoring was 3.5 ± 0.84 days, with 728 ± 202 glucose recordings per person. During CGMS monitoring, blood glucose levels were checked by finger-prick at least four times per day (before each meal and at bedtime).

Glycemic control was performed by the clinician. Intravenous insulin infusion with a micropump was performed as the first-line glucose-lowering treatment (Novolin R, Novo Nordisk, Bagsværd, Denmark). The protocol was as follows: Novolin R 50 IU + saline 50 ml, 4 ml/h intravenous injections with speed adjusted to maintain blood glucose levels in the range of 8–10 mmol/L. Sulfonylureas and α-glucosidase inhibitors were also used in some patients as a supplement. Other types of drugs, such as sodium-glucose cotransporter-2 (SGLT2) inhibitors, which have potential neuroprotective roles were not prescribed in these patients. Data of patients monitored for <48 h due to early death or due to sensor dropping were excluded.

Time above range (TAR) was defined as the percentage of time that blood glucose exceeded 10 mmol/L (180 mg/dL) during the entire glucose monitoring period for each patient. Hypoglycemic range was defined as the percentage of time that blood glucose was <3.9 mmol/L (70 mg/dL). Time in range (TIR) was defined as the percentage of time that a patient's blood glucose was between 3.9 and 10 mmol/L. Metrics of glycemic variation, including coefficient of variation (CV), standard deviation (SD), largest amplitude of glycemic excursion (LAGE), mean amplitude of glycemic excursion (MAGE), high blood glucose index (HBGI), low blood glucose index (LBGI), mean of daily difference (MODD), and % in hypoglycemic ranges, were also calculated from the CGMS data (17).

### Laboratory Tests

Blood glucose levels were assessed on admission. Serum levels of fasting blood glucose (FBG), 2-h post-prandial blood glucose (2-h PBG), hemoglobin A1c, C-reactive protein (CRP), hemocyanin (HCY), interleukin-1β (IL-1β), interleukin-2 receptor (IL-2R), interleukin-6 (IL-6), interleukin-8 (IL-8), interleukin-10 (IL-10), and tumor necrosis factor-α (TNF-α) were measured on the morning after admission.

### Statistical Analyses

Data are presented as frequencies (percentages) for categorical variables and as median (interquartile range) or mean ± SD for continuous variables. The Kolmogorov–Smirnov test was used to test the normality of data distribution. Univariable logistic regression analyses were used to test for associations between predictor and outcome variables. Variables with significant associations (*p* <0.05) were included in multivariate backward stepwise regression analysis. Forward selection was also used to confirm the robustness of multivariable confounders. Goodness of fit was assessed using the Hosmer–Lemeshow test. Correlations between inflammatory factors and glycemic variation were determined using Spearman's analysis. A *p-*value < 0.05 was considered statistically significant. All statistical analyses were performed using the Statistical Package for the Social Sciences version 19.0 for Windows (IBM Corporation, Armonk, NY, USA).

## Results

### Clinical Characteristics

**Figure 1:**
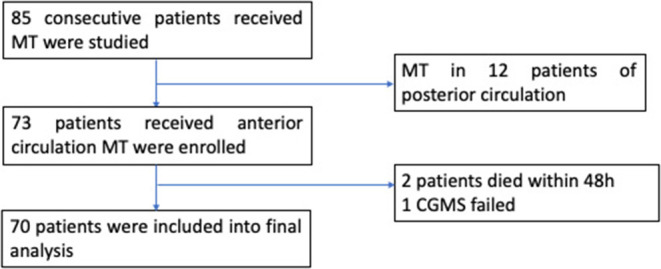
Flowchart of this study.

Of the 85 consecutive patients who underwent MT, 12 patients with posterior circulation AIS were excluded. Additionally, two patients died within 48 h after MT due to malignant cerebral infarction and hernia formation. One patient received CGMS device implantation, but data recording was incomplete (Figure 1). Finally, 70 patients (mean age: 72 ± 11 years, 38.6% female) were included in the analyses. The baseline characteristics of the patients are listed in Table 1.

**Table 1:** Clinical characteristics of patients with acute ischemic stroke (AIS) after mechanical thrombectomy (MT) (*N* = 70).

**Variable**	**Overall**
Age (years) mean ± SD	72 ± 11
Female sex, *n* (%)	27 (38.6%)
BMI (kg/m^2^), mean ± SD	23.89 ± 3.59
Smoke, *n* (%)	11 (15.7%)
Diabetes mellitus, *n* (%)	16 (22.9%)
Hypertension, *n* (%)	43 (61.4%)
Atrial fibrillation, *n* (%)	37 (52.9%)
HCY, μmol/L, median (IQR)	13.18 (10.13, 18.32)
CRP, mg/L, median (IQR)	6.88 (2.57, 31.26)
BG on admission, mmol/L, median (IQR)	7.3 (6.2, 8.8)
FBG, mmol/L, median (IQR)	7.17 (5.77, 9.15)
HbA1c, %	5.9 (5.5, 6.8)
NIHSS on admission, point, median (IQR)	18 (13, 21)
NIHSS at 24 h, points, median (IQR)	12 (5, 20)
NIHSS at 7 days, points, median (IQR)	7 (0, 15)
Occluded artery
ICA, *n* (%)	21 (30%)
M1, *n* (%)	40 (57.1%)
M2, *n* (%)	9 (12.9)
TOAST
LAA, *n* (%)	21 (30%)
CE, *n* (%)	40 (57.1%)
SUE, *n* (%)	9 (12.9%)
ORT, min, median (IQR)	294 (224, 360)
mTICI
2b or 3, *n* (%)	54 (77.1%)
sICH, *n* (%)	8 (11.4%)
Neurological improvement, *n* (%)	44 (62.9%)
In-hospital mortality, *n* (%)	10 (14.3%)
3-month mRS score, median (IQR)	3 (1, 5)
3-month functional independence, *n* (%)	28 (40%)
3-month excellent functional outcome, *n* (%)	20 (28.6%)
3-month mortality, *n* (%)	14 (20%)

*HCY, hemocyanin; CRP, C-reactive protein; FBG, fasting blood glucose; ICA, internal carotid artery; LAA, large artery atherosclerosis; CE, cardioembolism; SUE, stroke of undetermined cause; NIHSS, National Institute of Health Stroke Scale; mTICI, modified thrombolysis in cerebral infarction score; sICH, symptomatic intracerebral hemorrhage; ORT, onset to recanalization time; IQR, interquartile range*.

### Glycemic Variation and Outcomes of Patients With AIS After MT

Ten metrics were calculated using the CGMS data, percentages of TAR, hypoglycemic range, and TIR, CV, SD, MODD, MAGE, LAGE, HBGI, and LBGI. Glycemic variation metrics were treated as continuous variables. None of the metrics were found to be associated with the early neurological improvement and functional independence at the 3-month follow-up (Table 2). In the univariate analyses, older age, higher NIHSS scores at 24 h, higher LAGE, higher SD, and higher CV were associated with a greater likelihood of mortality in hospital and after discharge (Table 3).

**Table 2:** Univariable logistic regression analyses depicting the associations of glycemic variation indices with different functional outcomes.

**Variable**	**HYPO**	**TIR**	**TAR**	**CV**	**SD**	**LAGE**	**MAGE**	**MODD**	**LBGI**	**HBGI**
	**Odds ratio (95% CI)**									
Neurological improvement	2.032 (0.590, 6.997)	1.005 (0.991, 1.019)	0.994 (0.979, 1.009)	0.971 (0.904, 1.004)	0.713 (0.423, 1.203)	0.943 (0.826, 1.076)	1.027 (0.840, 1.256)	0.717 (0.466, 1.103)	1.177 (0.848, 1.634)	0.964 (0.906, 1.026)
3-Month functional independence	0.892 (0.542, 1.466)	0.986 (0.970, 1.001)	1.014 (0.997, 1.031)	1.019 (0.947, 1.096)	1.521 (0.845, 2.739)	1.047 (0.913, 1.200)	1.090 (0.886, 1.341)	1.325 (0.835, 2.102)	0.840 (0.643, 1.099)	1.077 (0.989, 1.174)
3-Month excellent functional outcome	0.959 (0.567, 1.624)	0.989 (0.972, 1.006)	1.010 (0.992, 1.029)	0.999 (0.924, 1.079)	1.348 (0.717, 2.535)	1.032 (0.890, 1.197)	1.085 (0.861, 1.366)	1.229 (0.747, 2.020)	1.035 (0.779, 1.374)	1.057 (0.966, 1.157)

*CI, confidence interval; HBGI, high blood glucose index; LBGI, low blood glucose index; MAGE, mean amplitude of glucose excursions; LAGE, largest amplitude of glycemic excursion; MODD, mean of daily differences; SD, standard deviation; CV, coefficient of variation; TIR, time in range; HYPO, hypoglycemic range; TAR, time above range*.

**Table 3:** Univariable logistic regression analysis depicting the associations of glycemic variation metrics, baseline characteristics with the likelihood of in-hospital and 3-month mortality.

**Variable**	**In-hospital mortality**		**3-Month mortality**	
	**Odds ratio (95% CI)**	***P***	**Odds ratio (95% CI)**	***P***
Age	1.093 (1.007, 1.186)	0.034	1.131 (1.041, 1.229)	0.004
Sex	0.359 (0.091, 1.415)	0.143	0.385 (0.117, 1.271)	0.117
BMI	0.963 (0.795, 1.165)	0.697	1.022 (0.862, 1.211)	0.804
Smoking	0.556 (0.063, 4.889)	0.596	0.354 (0.041, 3.025)	0.343
Diabetes mellitus	1.549 (0.351, 6.844)	0.563	0.902 (0.218, 3.730)	0.887
Hypertension	0.932 (0.237, 3.662)	0.920	1.742 (0.486, 6.241)	0.394
Atrial fibrillation	1.403 (0.359, 5.482)	0.626	1.800 (0.536, 6.050)	0.342
HCY	1.021 (0.964, 1.081)	0.476	1.040 (0.988, 1.095)	0.136
CRP	1.003 (0.991, 1.016)	0.606	1.010 (0.998, 1.022)	0.121
BG on admission	1.006(0.829, 1.221)	0.951	1.096 (0.993, 1.210)	0.068
FBG	1.015 (0.839, 1.228)	0.879	1.000 (0.842, 1.187)	0.997
HbA1c	0.963 (0.610, 1.521)	0.871	0.881 (0.564, 1.375)	0.577
NIHSS on admission	1.099 (0.985, 1.225)	0.090	1.096 (0.993, 1.210)	0.068
NIHSS at 24 h	1.092 (1.021, 1.167)	0.01	1.103 (1.036, 1.176)	0.002
Occluded artery				
ICA	0.359 (0.091, 1.415)	0.143	0.385 (0.117, 1.271)	0.117
MCA				
TOAST				
LAA	**Reference**		**Reference**	
CE	0.175 (0.014, 2.241)	0.18	6.667 (0.791, 56.215)	0.081
SUE	0.742 (0.126, 4.361)	0.742	10 (0.871, 114.746)	0.064
ORT	0.997 (0.992, 1.002)	0.243	0.996 (0.991, 1.000)	0.073
mTICI (2b or 3)	0.375 (0.091, 1.543)	0.174	0.290 (0.082, 1.022)	0.054
TAR (≥10 mmol/L)	1.014 (0.995, 1.034)	0.154	1.009 (0.990, 1.028)	0.336
TIR (3.9–10 mmol/L)	0.987 (0.969, 1.005)	0.160	0.992 (0.976, 1.009)	0.360
CV (%)	1.145 (1.036, 1.265)	0.008	1.093 (1.004, 1.190)	0.040
MAGE	1.260 (0.997, 1.598)	0.053	1.208 (0.971, 1.503)	0.089
LAGE	1.246 (1.051, 1.476)	0.011	1.163 (1.000, 1.352)	0.049
SD	2.697 (1.366, 5.325)	0.004	2.105 (1.156, 3.832)	0.015
MODD	1.433 (0.877, 2.342)	0.151	1.299 (0.823, 2.049)	0.261
Hypo-level range (%)	0.353 (0.018, 6.692)	0.490	0.233 (0.008, 6.857)	0.399
LBGI	0.814 (0.450, 1.473)	0.496	0.639 (0.278, 1.467)	0.291
HBGI	1.057 (0.986, 1.133)	0.117	1.036 (0.971, 1.105)	0.283

*CI, confidence interval; HBGI, high blood glucose index; LBGI, low blood glucose index; MAGE, mean amplitude of glucose excursions; LAGE, largest amplitude of glycemic excursion; MODD, mean of daily differences; SD, standard deviation; CV, coefficient of variation; TIR, time in range; HYPO, hypoglycemic range; TAR, time above range; HCY, hemocyanin; CRP, C-reactive protein; FBG, fasting blood glucose; ICA, internal carotid artery; LAA, large artery atherosclerosis; CE, cardioembolism; SUE, stroke of undetermined cause; NIHSS, National Institute of Health Stroke Scale; mTICI, modified thrombolysis in cerebral infarction score; sICH, symptomatic intracerebral hemorrhage; ORT, onset to recanalization time*.

In multivariable models using a backward-stepwise selection procedure, a higher SD was an independent risk factor for mortality, either in hospital (OR 2.8, 95% CI: 1.276–6.145, *p* = 0.010) or at the 3-month follow-up (OR 2.107, 95% CI: 1.013–4.382, *p* = 0.046). Older age and higher NIHSS at 24 h were also associated with a greater likelihood of 3-month mortality (Table 4). The forward-stepwise selection procedure was also used, and it confirmed the robustness of the multivariable model.

**Table 4:** Multiple logistic regression analysis depicting the associations of glycemic variation metrics, baseline characteristics with the likelihood of in-hospital and 3- month mortality.

**Variable**	**In-hospital mortality**		**3-Month mortality**	
	**Odds ratio (95% CI)**	***P***	**Odds ratio (95% CI)**	***P***
Age	1.094 (0.982, 1.218)	0.102	1.141 (1.028, 1.226)	0.013
NIHSS at 24 h	1.081 (1.000, 1.169)	0.050	1.092 (1.016, 1.174)	0.016
SD	2.800 (1.276, 6.145)	0.010	2.107 (1.013, 4.382)	0.046

*Data are OR (95% CI). Age, CV, SD, LAGE, NIHSS at 24 h were included into the multiple logistic regression model*.

*CI, confidence interval; OR, odds ratio; CV, coefficient of variation; LAGE, largest amplitude of glycemic variation; NIHSS, National Institute of Health Stroke Scale; SD, standard deviation*.

### Systemic Inflammation

Systemic inflammatory factors were assessed to explore the inflammatory response of patients with AIS after MT. The associations of blood glucose with systemic inflammatory factors, such as TNF-α, IL-6, IL-8, IL-1β, IL-2 receptors, and IL-10 in the serum of patients with LVO-AIS after MT were determined. There was a strong correlation of IL-10 with SD (Rs = 0.339 *p* = 0.026) and TNF-α and IL-8 levels with CV (TNF-α: Rs = 0.412, *p* = 0.006; IL-8: Rs = 0.430, *p* = 0.004). No association of glycemic variation expressed as SD or CV was found with IL-6 and IL-2 receptors (Table 5).

**Table 5:** Correlations between inflammatory factors and glycemic variation in patients with large vessel occlusion induced AIS (LVO-AIS).

	**SD**		**CV**	
	***R*_s_**	***P*-value**	***R*_s_**	***P*-value**
TNF-α	0.277	0.072	0.412	0.006
IL-6	0.191	0.231	0.191	0.232
IL-8	0.259	0.093	0.43	0.004
IL-1β	0.011	0.946	0.15	0.336
IL-2R	0.175	0.262	0.241	0.183
IL-10	0.339	0.026	0.222	0.152

*SD, standard deviation; CV, coefficient of variation; TNF, tumor necrosis factor; IL, interleukin*.

## Discussion

In this study, we demonstrated a strong association of increased systemic glycemic variation with a higher likelihood of mortality in patients with LVO-AIS who underwent MT.

MT is accepted as the first-line treatment for selected LVO–AIS of the anterior circulation to improve functional outcomes, as compared with standard medical therapy alone (2, 5, 18). However, nearly half of the patients present severe disability and one-fifth of the patients died after successful recanalization (6). In line with these reports, this study showed a high risk of in-hospital mortality of AIS after MT. Since many clinical characteristics may contribute to short-term mortality (19), it is essential to optimize critical care strategy for patients with AIS to improve the final outcome after recanalization. Dysglycemia is an important risk factor for poor outcomes in patients with AIS. Systemic glycemic variation was associated with mortality in some critically ill patient populations (20, 21). An early study using CGMS showed that increased glycemic variation was associated with the growth of infarction (22). Other groups showed that high glycemic variation in patients with AIS after MT correlated strongly with sICH (12) and poor functional outcomes at the 3-month follow-up (11). In this study, we showed similar results, in that increased glycemic variation, expressed as higher SD, was an independent risk factor for mortality in patients with AIS who had undergone MT (16).

Brain ischemia induces dysfunction of the periphery and brain immunity (23). A large volume of evidence has shown that, after AIS, there is an interaction between the central nervous system and the immune system, and these changes could be associated with clinical events, including infection, poor functional clinical outcomes, and mortality (24). Systemic inflammatory responses increase futile recanalization as well as reperfusion injury, leading to poor outcomes despite complete recanalization (25–27). Local brain inflammation also develops after stroke and aggravates secondary brain injury by exacerbating blood–brain barrier damage, microvascular failure, brain edema, and oxidative stress, and by directly inducing neuronal cell death (28). Cytokines, including TNF-α, IL-1, IL-8, and IL-6, are key mediators of inflammatory changes after brain ischemia (29). Increased concentrations of IL-6 and TNF-α in the blood of individuals with ischemic stroke have been correlated with a larger area of cerebral ischemia and worse outcomes (30, 31). IL-6 plasma levels correlate with cerebral perfusion deficits and infarct sizes in stroke patients without associated infections (32). Acute inflammation associated with ischemic–reperfusion injury is partly caused by IL-1 β (33). Preclinical studies have indicated that the IL-2 receptor α-IL-2 complex regulates T-cell differentiation and promotes white matter repair after ischemic stroke (34). IL-10 is an antiinflammatory cytokine. The interaction of pro-inflammatory and antiinflammatory cytokines after stroke is not completely clear. Previous studies have shown that glucose fluctuation had a deleterious effect on endothelial function (35, 36) by enhancing oxidative stress (37–39) and producing pro-inflammatory cytokines (40). In this study, we found a correlation between glycemic variation and serum inflammatory factors in patients with AIS after reperfusion. This may be related to the adverse effect of glycemic variation on stroke outcomes depending on improper immune reactions.

Greater glycemic variation is likely correlated with a higher risk of hypoglycemic episodes. In this study, the moderate hypoglycemia level range was determined from CGMS recording but not by finger-prick glucose measurements. Asymptomatic hypoglycemic events need to be treated with medicine. We did not detect more hypoglycemic events in the group showing mortality than in the group who survived. Thus, we consider that higher SD is an independent risk factor of mortality, independent of hypoglycemic damage.

This study had some limitations. First, this was an observational study. We were not able to determine the causal role of high glycemic variation on mortality. We could not differentiate between a direct or an indirect relationship between glycemic variation and mortality. Second, we did not record the specific cause of death for each patient. We adopted composite death as the endpoint. Third, systemic inflammatory cytokines may exhibit temporal dynamic changes after reperfusion. Thus, serum should be collected at different time points following reperfusion, and a comprehensive profile of cytokines related to glycemic variation should be established. Lastly, the sample size was too small to determine the association of glycemic variation with functional outcomes.

In conclusion, this study demonstrated that increased glycemic variation in patients with LVO–AIS of the anterior circulation is significantly associated with a higher risk of mortality. Inflammatory factors may play a role in the association of glycemic variation with mortality. Higher glycemic variation not only predicts mortality but may also be a target for decreasing mortality in future clinical practice. Another translational study aiming to evaluate the impact of glycemic variation on AIS outcomes in humans is ongoing (41). Further, additional large-scale studies are required to explore the role of glycemic variation lowering treatment on the long-term prognosis of LVO–AIS after MT.

## Data Availability Statement

The original contributions presented in the study are included in the article/supplementary material, further inquiries can be directed to the corresponding authors.

## Ethics Statement

The studies involving human participants were reviewed and approved by Ethics Committee of Shanghai Jiao Tong University Affiliated Sixth People's Hospital. The patients/participants provided their written informed consent to participate in this study.

## Author Contributions

JD and YZ were involved in design of the study. JD wrote the manuscript. LL and FC collected all the data. FW and HW evaluated the results. LS was involved in the statistical analysis. FZ and HS evaluated results and revised the manuscript. All authors have approved the final version of the manuscript.

## Funding

This research was supported by a grant from the Shanghai Municipal Health Commission (201840099), the National Natural Science Youth Foundation of China (82001303), and the Science and Technology Commission of Shanghai Municipality (19411968500).

## Conflict of Interest

The authors declare that the research was conducted in the absence of any commercial or financial relationships that could be construed as a potential conflict of interest.

## Publisher's Note

All claims expressed in this article are solely those of the authors and do not necessarily represent those of their affiliated organizations, or those of the publisher, the editors and the reviewers. Any product that may be evaluated in this article, or claim that may be made by its manufacturer, is not guaranteed or endorsed by the publisher.

## References

[1] Feigin VL Nguyen G Cercy K Johnson CO Alam T Parmar PG. Global, regional, and country-specific lifetime risks of stroke, 1990 and 2016. N Engl J Med. (2018) 379:2429–37. 10.1056/NEJMoa1804492 30575491 PMC6247346

[2] Powers WJ Rabinstein AA Ackerson T Adeoye OM Bambakidis NC Becker K. Guidelines for the early management of patients with acute ischemic stroke: 2019 update to the 2018 guidelines for the early management of acute ischemic stroke: a guideline for healthcare professionals from the American Heart Association/American Stroke Association. Stroke. (2019) 50:e344–418. 10.1161/STR.0000000000000211 31662037

[3] Campbell BCV De Silva DA Macleod MR Coutts SB Schwamm LH Davis SM. Ischaemic stroke. Nat Rev Dis Primers. (2019) 5:70. 10.1038/s41572-019-0118-8 31601801

[4] Phipps MS Cronin CA. Management of acute ischemic stroke. BMJ. (2020) 368:l6983. 10.1136/bmj.l6983 32054610

[5] Goyal M Menon BK van Zwam WH Dippel DW Mitchell PJ Demchuk AM. Endovascular thrombectomy after large-vessel ischaemic stroke: a meta-analysis of individual patient data from five randomised trials. Lancet. (2016) 387:1723–31. 10.1016/S0140-6736(16)00163-X 26898852

[6] Jahan R Saver JL Schwamm LH Fonarow GC Liang L Matsouaka RA. Association between time to treatment with endovascular reperfusion therapy and outcomes in patients with acute ischemic stroke treated in clinical practice. JAMA. (2019) 322:252–63. 10.1001/jama.2019.8286 31310296 PMC6635908

[7] Uyttenboogaart M Koch MW Stewart RE Vroomen PC Luijckx GJ De Keyser J. Moderate hyperglycaemia is associated with favourable outcome in acute lacunar stroke. Brain. (2007) 130:1626–30. 10.1093/brain/awm087 17525141

[8] Kruyt ND Nys GM van der Worp HB van Zandvoort MJ Kappelle LJ Biessels GJ. Hyperglycemia and cognitive outcome after ischemic stroke. J Neurol Sci. (2008) 270:141–7. 10.1016/j.jns.2008.02.020 18387635

[9] Johnston KC Bruno A Pauls Q Hall CE Barrett KM Barsan W. Intensive vs standard treatment of hyperglycemia and functional outcome in patients with acute ischemic stroke: the SHINE randomized clinical trial. JAMA. (2019) 322:326–35. 10.1001/jama.2019.9346 31334795 PMC6652154

[10] Camara-Lemarroy CR. Glucose and stroke: what about glycemic variability? J Neurol Sci. (2017) 373:242–3. 10.1016/j.jns.2017.01.015 28131196

[11] Gordon WR Salamo RM Behera A Chibnall J Alshekhlee A Callison RC. Association of blood glucose and clinical outcome after mechanical thrombectomy for acute ischemic stroke. Interv Neurol. (2018) 7:182–8, 10.1159/000486456 29719556 PMC5921213

[12] Kim TJ Lee JS Park SH Ko SB. Short-term glycemic variability and hemorrhagic transformation after successful endovascular thrombectomy. Transl Stroke Res. (2021) 12:968–75. 10.1007/s12975-021-00895-4 33576937

[13] Warner JJ Harrington RA Sacco RL Elkind MSV. Guidelines for the early management of patients with acute ischemic stroke: 2019 update to the 2018 guidelines for the early management of acute ischemic stroke. Stroke. (2019) 50:3331–2. 10.1161/STROKEAHA.119.027708 31662117

[14] Hacke W Kaste M Fieschi C von Kummer R Davalos A Meier D. Randomised double-blind placebo-controlled trial of thrombolytic therapy with intravenous alteplase in acute ischaemic stroke (ECASS II). Second European-Australasian Acute Stroke Study Investigators. Lancet. (1998) 352:1245–51. 10.1016/S0140-6736(98)08020-9 9788453

[15] Luitse MJ Biessels GJ Rutten GE Kappelle LJ. Diabetes, hyperglycaemia, and acute ischaemic stroke. Lancet Neurol. (2012) 11:261–71. 10.1016/S1474-4422(12)70005-4 22341034

[16] Palaiodimou L Lioutas VA Lambadiari V Theodorou A Themistocleous M Aponte L. Glycemic variability of acute stroke patients and clinical outcomes: a continuous glucose monitoring study. Ther Adv Neurol Disord. (2021) 14:17562864211045876. 10.1177/17562864211045876 34589140 PMC8474316

[17] Monnier L Colette C Owens DR. The application of simple metrics in the assessment of glycaemic variability. Diabetes Metab. (2018) 44:313–9. 10.1016/j.diabet.2018.02.008 29602622

[18] Hussain M Moussavi M Korya D Mehta S Brar J Chahal H. systematic review and pooled analyses of recent neurointerventional randomized controlled trials: setting a new standard of care for acute ischemic stroke treatment after 20 years. Interv Neurol. (2016) 5:39–50. 10.1159/000442355 27610120 PMC4934481

[19] Chen CJ Chuang TY Hansen L Dutta S Ding D Buell TJ. Predictors of 30-day mortality after endovascular mechanical thrombectomy for acute ischemic stroke. J Clin Neurosci. (2018) 57:38–42. 10.1016/j.jocn.2018.08.044 30145087

[20] Kurtz P Claassen J Helbok R Schmidt J Fernandez L Presciutti M. Systemic glucose variability predicts cerebral metabolic distress and mortality after subarachnoid hemorrhage: a retrospective observational study. Crit Care. (2014) 18:R89. 10.1186/cc13857 24887049 PMC4056693

[21] Ma H Yu G Wang Z Zhou P Lv W. Association between dysglycemia and mortality by diabetes status and risk factors of dysglycemia in critically ill patients: a retrospective study. Acta Diabetol. (2021). 10.1007/s00592-021-01818-3. [Epub ahead of print]. 34761326 PMC8917030

[22] Shimoyama T Kimura K Uemura J Saji N Shibazaki K. Post stroke dysglycemia and acute infarct volume growth: a study using continuous glucose monitoring. Eur Neurol. (2016) 76:167–74. 10.1159/000448329 27643995

[23] Liu Q Jin WN Liu Y Shi K Sun H Zhang F. Brain ischemia suppresses immunity in the periphery and brain via different neurogenic innervations. Immunity. (2017) 46:474–87. 10.1016/j.immuni.2017.02.015 28314594

[24] Iadecola C Anrather J. The immunology of stroke: from mechanisms to translation. Nat Med. (2011) 17:796–808. 10.1038/nm.2399 21738161 PMC3137275

[25] Lattanzi S Norata D Divani AA Di Napoli M Broggi S Rocchi C. Systemic inflammatory response index and futile recanalization in patients with ischemic stroke undergoing endovascular treatment. Brain Sci. (2021) 11:1164. 10.3390/brainsci11091164 34573185 PMC8468021

[26] Chen Z He Y Su Y Sun Y Zhang Y Chen H. Association of inflammatory and platelet volume markers with clinical outcome in patients with anterior circulation ischaemic stroke after endovascular thrombectomy. Neurol Res. (2021) 43:503–10. 10.1080/01616412.2020.1870359 33402058

[27] Xu X Yuan L Wang W Xu J Yang Q Zhu Y. Systemic inflammatory response syndrome and outcomes in ischemic patients treated with endovascular treatment. Clin Interv Aging. (2020) 15:2331–40, 10.2147/CIA.S281865 33324045 PMC7733387

[28] Shi K Tian DC Li ZG Ducruet AF Lawton MT Shi FD. Global brain inflammation in stroke. Lancet Neurol. (2019) 18:1058–66. 10.1016/S1474-4422(19)30078-X 31296369

[29] Pluta R Januszewski S Czuczwar SJ. Neuroinflammation in post-ischemic neurodegeneration of the brain: friend, foe, or both? Int J Mol Sci. (2021) 22:4405. 10.3390/ijms22094405 33922467 PMC8122836

[30] Mazzotta G Sarchielli P Caso V Paciaroni M Floridi A Floridi A. Different cytokine levels in thrombolysis patients as predictors for clinical outcome. Eur J Neurol. (2004) 11:377–81. 10.1111/j.1468-1331.2004.00798.x 15171733

[31] Beridze M Sanikidze T Shakarishvili R Intskirveli N Bornstein NM. Selected acute phase CSF factors in ischemic stroke: findings and prognostic value. BMC Neurol. (2011) 11:41. 10.1186/1471-2377-11-41 21450100 PMC3078848

[32] Hotter B Hoffmann S Ulm L Meisel C Fiebach JB Meisel A. IL-6 plasma levels correlate with cerebral perfusion deficits and infarct sizes in stroke patients without associated infections. Front Neurol. (2019) 10:83. 10.3389/fneur.2019.00083 30828313 PMC6384225

[33] Wanderer AA. Ischemic-reperfusion syndromes: biochemical and immunologic rationale for IL-1 targeted therapy. Clin Immunol. (2008) 128:127–32. 10.1016/j.clim.2008.03.514 18479971

[34] Shi L Sun Z Su W Xu F Xie D Zhang Q. Treg cell-derived osteopontin promotes microglia-mediated white matter repair after ischemic stroke. Immunity. (2021) 54:1527–42.e8. 10.1016/j.immuni.2021.04.022 34015256 PMC8282725

[35] Ceriello A Esposito K Piconi L Ihnat MA Thorpe JE Testa R. Oscillating glucose is more deleterious to endothelial function and oxidative stress than mean glucose in normal and type 2 diabetic patients. Diabetes. (2008) 57:1349–54. 10.2337/db08-0063 18299315

[36] Wu N Shen H Liu H Wang Y Bai Y Han P. Acute blood glucose fluctuation enhances rat aorta endothelial cell apoptosis, oxidative stress and pro-inflammatory cytokine expression *in vivo*. Cardiovasc Diabetol. (2016) 15:109. 10.1186/s12933-016-0427-0 27496150 PMC4974767

[37] Quagliaro L Piconi L Assaloni R Martinelli L Motz E Ceriello A. Intermittent high glucose enhances apoptosis related to oxidative stress in human umbilical vein endothelial cells: the role of protein kinase C and NAD(P)H-oxidase activation. Diabetes. (2003) 52:2795–804. 10.2337/diabetes.52.11.2795 14578299

[38] Ge QM Dong Y Zhang HM Su Q. Effects of intermittent high glucose on oxidative stress in endothelial cells. Acta Diabetol. (2010) 47(Suppl. 1):97–103. 10.1007/s00592-009-0140-5 19763390

[39] Monnier L Mas E Ginet C Michel F Villon L Cristol JP. Activation of oxidative stress by acute glucose fluctuations compared with sustained chronic hyperglycemia in patients with type 2 diabetes. JAMA. (2006) 295:1681–7. 10.1001/jama.295.14.1681 16609090

[40] Quagliaro L Piconi L Assaloni R Da Ros R Maier A Zuodar G. Intermittent high glucose enhances ICAM-1, VCAM-1 and E-selectin expression in human umbilical vein endothelial cells in culture: the distinct role of protein kinase C and mitochondrial superoxide production. Atherosclerosis. (2005) 183:259–67. 10.1016/j.atherosclerosis.2005.03.015 16285992

[41] Fuentes B Pastor-Yborra S Gutiérrez-Zúñiga R González-Pérez de Villar N de Celis E Rodríguez-Pardo J. Glycemic variability: prognostic impact on acute ischemic stroke and the impact of corrective treatment for hyperglycemia. The GLIAS-III translational study. J Transl Med. (2020) 18:414. 10.1186/s12967-020-02586-4 33148277 PMC7610240

